# Grazing on Marine Viruses and Its Biogeochemical Implications

**DOI:** 10.1128/mbio.01921-21

**Published:** 2023-01-30

**Authors:** Kyle M. J. Mayers, Constanze Kuhlisch, Jonelle T. R. Basso, Marius R. Saltvedt, Alison Buchan, Ruth-Anne Sandaa

**Affiliations:** a Environment and Climate Division, NORCE Norwegian Research Centre, Bergen, Norway; b Department of Plant and Environmental Sciences, Weizmann Institute of Science, Rehovot, Israel; c Department of Microbiology, University of Tennessee Knoxville, Knoxville, Tennessee, USA; d Department of Microbiology, University of Bergen, Bergen, Norway; Albert Einstein College of Medicine

**Keywords:** marine viruses, *Nucleocytoviricota*, grazing, biogeochemistry, macronutrients, micronutrients

## Abstract

Viruses are the most abundant biological entities in the ocean and show great diversity in terms of size, host specificity, and infection cycle. Lytic viruses induce host cell lysis to release their progeny and thereby redirect nutrients from higher to lower trophic levels. Studies continue to show that marine viruses can be ingested by nonhost organisms. However, not much is known about the role of viral particles as a nutrient source and whether they possess a nutritional value to the grazing organisms. This review seeks to assess the elemental composition and biogeochemical relevance of marine viruses, including roseophages, which are a highly abundant group of bacteriophages in the marine environment. We place a particular emphasis on the phylum *Nucleocytoviricota* (NCV) (formerly known as nucleocytoplasmic large DNA viruses [NCLDVs]), which comprises some of the largest viral particles in the marine plankton that are well in the size range of prey for marine grazers. Many NCVs contain lipid membranes in their capsid that are rich carbon and energy sources, which further increases their nutritional value. Marine viruses may thus be an important nutritional component of the marine plankton, which can be reintegrated into the classical food web by nonhost organism grazing, a process that we coin the “viral sweep.” Possibilities for future research to resolve this process are highlighted and discussed in light of current technological advancements.

## INTRODUCTION

Viruses are important drivers of evolution, population dynamics, and biodiversity in the marine environment (1, 2). Furthermore, they influence the fluxes of nutrients, organic matter, and energy (3, 4). Seawater is teeming with viruses, reaching up to 6.5 × 10^7^ viral particles per mL of water (5), leading to the infection and death of a diverse range of marine microbes, including bacteria and protists (single-celled eukaryotes), as well as macroorganisms, such as fish and mollusks (6). Viral infections, particularly by lytic viruses, can bring about subsequent cell lysis, causing an array of chemically and structurally diverse molecules within cells to be released into the surrounding environment. As a result, viruses play a major role in the cycling of biogeochemical elements, such as carbon (C), nitrogen (N), and phosphorus (P), within marine ecosystems (7–9). Most notably, the process of cell lysis diverts organic material away from the classical food web (i.e., the carbon flux from primary producers to predators), a process known as the “viral shunt” (4, 9). The effect of this short-circuit is increased C availability for respiration by bacteria and other heterotrophic microorganisms. Conversely, the lysis and release of sticky transparent exopolymeric particles (TEPs) can lead to enhanced sinking of organic material from upper to lower oceanic layers, known as the “viral shuttle” (10, 11). Also, lysogenic infections by temperate phages can occur, which likely lead to long-term associations with the respective host and modulation of host physiology (e.g., morphology, gene expression, and metabolism) (12, 13). Although the presence and influence of lysogenic infections are significant in marine systems (14–16), lysogeny itself is very complex and little is known about the induction of the lytic form. Therefore, the direct contribution of temperate phages as free viral particles for marine grazing and nutrient cycling is currently unknown, but they will indirectly have an influence by altering host physiology. By influencing the availability and cycling of major elements and nutrients, viruses clearly play important roles in the structuring and functioning of marine ecosystems.

Growth and biomass production within marine ecosystems are regulated by many factors, including the availability and concentration of macronutrients (e.g., C, N, and P) and micronutrients (e.g., iron [Fe] and zinc). Macronutrients are the primary elements in the building blocks of every living cell, namely, DNA, proteins, and lipid membranes. Carbon can be fixed photoautotrophically by phytoplankton in the sunlit upper ocean layers, which is the primary pathway for C and energy to enter the marine food web, supporting the growth of diverse heterotrophic organisms (17). As marine microbes grow, they assimilate dissolved C, N, and P from their environment, which can lead to a depletion of these macronutrients, most notably N and P. Within coastal marine environments, it is typical that N limits microbial growth at certain times of the year, while in freshwater environments, the Mediterranean Sea, and tropical ocean waters, P is typically the primary limiting nutrient (18–21). In addition, micronutrients, particularly Fe, frequently limit the growth of marine microbes (22). In the Fe-limited regions of the Southern Ocean, new production is suggested to be largely driven by Fe recycling (23). Viruses thereby can have a major contribution to Fe recycling, as lytic viral infection leads to the release of host cell constituents that are rich in bioavailable Fe (24).

Marine microorganisms have evolved several adaptations to circumvent nutrient limitation that give them the ability to access and utilize various limiting compounds, which in turn affords them a competitive advantage under nutrient-limiting conditions (20). By feeding on microbes, heterotrophic predators (e.g., zooplankton) are often more limited by the availability of nutrient-rich prey or detrital particles, rather than ambient nutrient concentrations themselves. Among predatory organisms, different feeding strategies exist, leading to variation in success in accessing potential food sources (25). For example, filter feeders, such as pelagic tunicates and pteropods, process large volumes of water to trap and ingest particles, which are sometimes several magnitudes smaller than the predators themselves (26). The ability to access and utilize the various components that exist in particulate form allows a competitive advantage and continued growth within nutrient-limited systems. Marine viruses, being the most abundant biological particles in the ocean (4), might thus be an important food source for heterotrophic organisms. However, their relevance as such and the extent of predation upon them are still enigmatic.

Due to their size, viral particles fall into the pool of organic matter that is frequently categorized as dissolved organic matter (DOM). This pool is operationally defined by filtration cutoffs, typically ranging between 0.2 and 0.7 μm (27). Inspired by a paper on the elemental stoichiometry and contribution of marine bacteriophages (viruses that infect bacteria) to the DOM pool by Jover et al. (28), we sought to further assess the question of the nutritional value and biogeochemical influence of marine viruses by applying their approach to marine giant viruses (eukaryotic viruses with particularly large genome and particle sizes). These viruses infect globally distributed marine plankton, can have genome and particle sizes that are comparable to those of bacteria, and can occur in abundances up to 10^6^ mL^−1^ (29, 30), thus likely contributing significantly to the DOM pool. Jover et al. used a biophysical scaling model to determine that while the contribution of bacteriophages to the dissolved organic carbon (DOC) pool may be low due to low C/N and C/P ratios, these particles can represent significant components of the dissolved organic N and P pools (DON and DOP, respectively) (28). Phosphorus might be of particular importance, as viruses can represent up to 8% of the total DOP in surface waters (28). As N- and P-rich particles, bacteriophages have the potential to be a valuable food source for heterotrophs and could further redirect macronutrients and energy back to higher trophic levels.

In this review, we summarize the current literature on compositional aspects of different viral particles that define their role as a valuable source for nutrients and energy, on grazing rates of marine viral particles, and on evidence for their digestion. To determine the biogeochemical impact of important virus groups in the sunlit ocean, we expand upon earlier efforts in modeling the elemental stoichiometry of bacteriophage particles (28) by comparing the previously assessed bacteriophages with recently sequenced ubiquitous and abundant roseophages as well as with the larger and structurally more complex giant viruses. Although lysogeny may indirectly affect grazing and biogeochemical cycles via the rewiring of host cell metabolism, in this review, we focus on lytic viral infections due to their release of viral particles that are directly available for marine grazers. For more in-depth commentaries on the different fates of viral particles in the ocean, including particle adsorption and degradation as well as the influence of environmental factors, such as UV light, the reader may refer to Mojica and Brussaard (31) and Zhang et al. (32).

## VIRUSES ARE GRAZED UPON BY DIVERSE MARINE ORGANISMS

The first observations that nonhost predators can ingest virus-like particles came from heterotrophic nanoflagellates, predators that are 2 to 20 μm in size and thus classified as microscopic. They were reported to be able to ingest viral particles of ~100 nm in size, clearing about 4% of the standing viral community in 1 day (33, 34). Recently, single-cell genomics has been applied to similarly sized protists in natural plankton communities, revealing that a significant proportion of nonhost cells contained viral DNA (35). This was observed in two contrasting environments, with a higher fraction of cells containing viral DNA in the Gulf of Maine (51%) than in the Mediterranean Sea (35%). Viral sequences were distributed nonrandomly across taxa and showed elevated numbers in specific individuals, suggesting that predation upon viral particles is the most likely cause of this observation, rather than virus cosorting and nonspecific attachment. Interestingly, for algae belonging to the picozoa and choanozoa lineages, 100% of the sorted cells contained viral DNA sequences. Both picozoa and choanozoa are known suspension feeders (36, 37), where particle ingestion is nonselective and related to a minimum prey size (25, 38), which indicates that these groups may be significant predators of viral particles in the marine environment.

Macroorganisms have also been observed to ingest viruses. The Red Sea sponge Negombata magnifica displayed an ability to filter viruses with a mean efficiency of 23% (39). As this study used flow cytometry to quantify the total *in situ* viral community, the size distribution of the consumed viruses was not evident. However, a more recent study using a large marine algal virus (Phaeocystis globosa *virus 07T* [PgV07T]; 160 nm in diameter) detected significant clearance by a bread crumb sponge (Halichondria panicea) (40). These results show that not only are different sponge species able to filter viruses of various sizes, but this is likely a general mechanism in tropical and temperate coastal areas, where sponges are frequently found. Based on the high abundance of sponges in these environments, their filtration rates with up to 35 mL min^−1^ sponge cm^−3^ accompanied by high retention efficiencies of small particles, and their importance in bentho-pelagic coupling (41, 42), sponges may be key players in removing viral particles from the water column in coastal areas and redirecting macronutrients to higher trophic levels.

Feeding of large algal viruses has also been observed in other organisms. Using PgV07T, Welsh et al. demonstrated that a number of zooplanktonic organisms could ingest this virus, including the larvae of littoral crabs (Carcinus maenas), oysters (Magallana gigas), and polychaetes (marine bristle worms [a mixture of species]) (40). Similarly, using another large algal virus (Emiliania huxleyi *virus 99B1* [EhV 99B1]; 180 nm in diameter), it was shown that the globally distributed pelagic (free-swimming) tunicate Oikopleura dioica was able to trap viruses at high rates, leading to almost 100% clearance of viral particles within ~1 day in a closed system (43). This was also shown for *O. dioica* feeding on natural viral communities (44). Many of the above-mentioned organisms are filter feeders, suggesting that viral grazing may be common to this feeding class of predators. Observed clearance of large viruses, with rates as high as 90.3 mL^−1^ day^−1^ individual^−1^ (see Table S1 in the supplemental material), by different organisms from both coastal and open ocean habitats suggests that nonhost grazing interactions may be widespread in marine environments, particularly when the large size range of marine viruses is considered (~120 to 400 nm in diameter).

10.1128/mbio.01921-21.2 TABLE S1 Reported grazing rates on viral particles by marine organisms. Download Table S1, DOCX file, 0.01 MB. Copyright © 2023 Mayers et al. 2023 Mayers et al. https://creativecommons.org/licenses/by/4.0/ This content is distributed under the terms of the Creative Commons Attribution 4.0 International license.

## VIRUSES AS A FOOD SOURCE—ARE SOME VIRUSES MORE NUTRITIOUS THAN OTHERS?

Although generally smaller than algal viruses, bacteriophages are estimated to be the most numerous viruses in the marine environment (45). Not only do they frequently outnumber their bacterial counterparts, they also significantly influence bacterial community dynamics and biogeochemistry (45, 46). On the other hand, marine bacterial community structure strongly influences the burst size of their associated bacteriophages, as well as bacteriophage production itself (47). Jover et al. found that bacteriophages can be significant contributors to DON, DOP, and, to a lesser extent, DOC (28). This is because bacteriophages mainly consist of DNA as well as proteinaceous tails and capsids, which are composed of molecules rich in N and P (48–50). An abundant and biogeochemically relevant group of bacteriophages are roseophages, which infect the ubiquitous *Roseobacteraceae* (51–53), which are commonly found in association with blooms of eukaryotic alga (52, 54). Of the 32 roseophages currently described (55), 19 are short tailed (podoviridae), 11 are of filamentous tail structure (siphoviridae), and two are single-stranded DNA bacteriophages. Here, we focus on the double-stranded DNA (dsDNA) roseophages only, which were shown to range in genome size from 35.9 kb (56) to 147.5 kb (55). Given their numerical abundance, roseophages can be a significant fraction of the DOM pool, and if ingested, could collectively be a significant source of nutrition.

Most marine eukaryotic viruses infecting algae and protozoans (57) belong to the phylum *Nucleocytoviricota* (NCV) (formerly known as nucleocytoplasmic large DNA viruses [NCLDVs]) (58). Using metagenomics, NCVs were revealed to be an abundant and widespread viral group (30). These viruses are dsDNA viruses that vary in their genome and capsid sizes. In general, NCVs are considered very large, with particle diameters ranging between 120 and 520 nm and genome sizes ranging between 173 and 1,573 kb (Table S2), which even exceed the sizes of some bacterial genomes (59). They are thus also often referred to as giant viruses. Unlike bacteriophages, many NCVs possess capsids of tremendous morphological complexity, including structures such as internal lipid membranes and external capsid fibers (60, 61). The lipid membranes of algal NCVs can occupy up to 66% of the interior of the viral capsid (Prymnesium kappa *virus RF01* [PkV RF01]) (62) and are composed of various lipid types, including C-rich triacylglycerides, P-rich phospholipids, and N-rich betaine lipids (63, 64). Some NCV capsids are further surrounded by an outer lipid membrane, as is the case in Emiliania huxleyi *virus 86* (65) and multiple virus strains infecting Micromonas pusilla (61). This structural complexity of NCV will ultimately influence their elemental stoichiometry and the nutritional value for predators.

**FIG 1:**
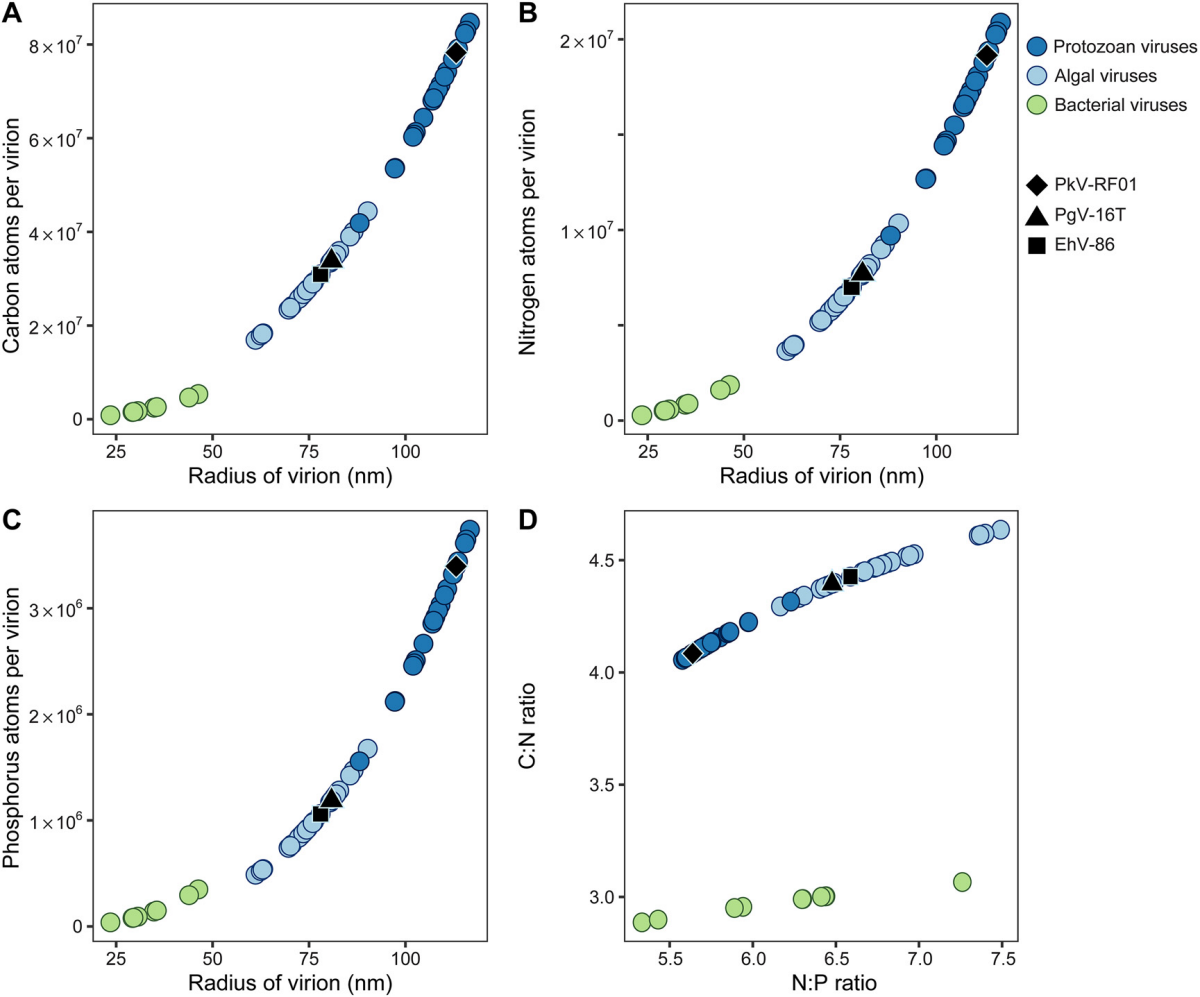
Elemental composition of aquatic giant viruses and bacteriophages. Atoms of carbon (A), nitrogen (B), and phosphorus (C) per virion were derived by a size-dependent model. Color determines whether the virus infects algae (light blue; *n *= 29), protozoans (dark blue; *n *= 24), or, for comparison, bacteria (green; *n *= 11). The elemental ratios of C/N and N/P are displayed (D). Modified calculations for marine and freshwater NCVs are displayed in comparison to calculations for marine bacteriophages that were done using the model described by Jover et al. (28). Black shapes display selected viruses for which feeding by marine grazers has been observed, including *Emiliania huxleyi virus 86* (EhV-86 [black squares]), *Phaeocystis globosa virus 16T* (PgV-16T [black triangles]), and *Prymnesium kappa virus RF01* (PkV-RF01 [black diamonds]). For a full list, see Table S2.

10.1128/mbio.01921-21.3 TABLE S2 Summary data on viruses used for viral C, N, and P contents modeled for Fig. 1. Download Table S2, XLSX file, 0.03 MB. Copyright © 2023 Mayers et al. 2023 Mayers et al. https://creativecommons.org/licenses/by/4.0/ This content is distributed under the terms of the Creative Commons Attribution 4.0 International license.

NCVs infect a wide range of eukaryotic hosts in diverse biogeographical regions (30, 66). Among these are also various single-cell organisms that, while they are usually present in low abundances in the photic zone, can develop seasonally high-density blooms (67). In these highly proliferative environments, viral infection can lead to the termination of the bloom-forming host population and to the release of large numbers of viral particles (29, 68). Although most NCVs seem to have a rather persistent abundance throughout the year (30, 69, 70), such “boom and bust” dynamics following host blooms may provide a temporarily abundant food source for the filter feeders among marine grazers. Furthermore, we posit that owing to their distinct structural characteristics, NCV particles can have a significant contribution to the marine DOM pool.

## THE BIOGEOCHEMICAL IMPACT OF VIRIONS ON THE MARINE ENVIRONMENT

Following the stoichiometric model developed by Jover et al., viral particles represent particularly N- and P-rich particles within the DOM pool (28). This model was developed to determine the elemental composition of bacteriophages to assess their contribution to the marine DOM pool. In short, this model assumes that the bacteriophage head is spherical (described by its external radius) and that a fraction of about 53% is filled with DNA. The bacteriophage capsid is described as a spherical shell with a uniform thickness of about 2.5 nm, thereby taking up a defined volume composed solely of proteins. A size-dependent scaling is defined based on the bacteriophage head radius, with DNA scaling to the cube of the radius and proteins scaling to the square of the radius. While the majority of bacteriophages have tails composed of proteins and DNA (71), the elemental stoichiometry was for simplicity modeled for the spherical bacteriophage head only. This parameterization was found to be appropriate for the roseophages exemplarily modeled in this review (see below). To emphasize the biogeochemical relevance of NCV in the marine environment and the need to improve our understanding of their contribution and fate within the DOM pool, we applied the model of Jover et al. for a selection of NCVs and adjusted the parameters in accordance with the current literature as described below.

NCVs have larger genomes with reduced DNA packaging densities in their virions compared to bacteriophages, possibly due to differences in their life histories (57). This is particularly apparent for NCVs infecting freshwater protozoa, such as *Marseilleviridae* and *Mimiviridae*, which have an average DNA content of 0.07 (equivalent to 7%). As the focus of this review is on the marine environment, we adjusted the filling fraction from 0.53 ± 0.04 for bacteriophages to 0.33 ± 0.16, accounting for the lower DNA volume fraction in marine algal NCVs, such as *Phycodnaviridae* (as described in references 72 to 77 and Text S1). Furthermore, many NCVs possess an inner lipid membrane, typically a bilayer, directly below the capsid protein shell (61, 62, 78, 79). We therefore increased the shell thickness to 10 nm following Chaudhari et al. (57). Finally, we aimed to include the frequent occurrence of lipid membranes in NCVs by defining a simplified elemental composition for these lipid membranes. It must be noted, however, that the current literature on the membrane composition of NCVs is scarce. We thus computed the elemental composition for a simple membrane bilayer with 0.5 nm^2^ surface area per lipid molecule (80), composed solely of phosphatidylcholines with fatty acid chain lengths of 16:0 and 16:1, which were reported to be the most abundant fatty acids in marine algae (81), thus assuming that the lipid composition of algal NCV resembles that of the host cell. For the full details on the original model for bacteriophages, the reader may refer to Jover et al. (28), and for the full details on the modifications for marine algal NCVs, the reader may refer to Text S1 of this review.

10.1128/mbio.01921-21.1 TEXT S1 Supplemental text including information about stoichiometric model adjustment calculation of clearance rates. Download Text S1, DOCX file, 0.02 MB. Copyright © 2023 Mayers et al. 2023 Mayers et al. https://creativecommons.org/licenses/by/4.0/ This content is distributed under the terms of the Creative Commons Attribution 4.0 International license.

The number of C, N, and P atoms in NCV scales with the radius of the viral particle (60 to 120 nm), leading to an elemental content that is 1 order of magnitude higher than that of bacteriophages, in part due to their size difference (Fig. 1A to C). The presence of an inner lipid membrane affects, in particular, the C/N ratio of the NCV particles (Fig. 1D). While the C/N ratio is ~3 for bacteriophages (28), NCVs are less enriched in N with C/N ratios of 4.1 to 4.6, which is closer to the C/N ratio of natural bacterial assemblages in marine environments (6.8 ± 1.2) (82). The N and P contents are primarily affected by the adjustment of the capsid-filling fraction for marine algal NCVs, leading to N/P ratios of 6.5 to 7.5 (Fig. 1D). Even higher N/P ratios for algal NCVs can be expected, assuming that some of the remaining volume is occupied by proteins that stabilize DNA (83, 84) or help kick-start translation upon infection (85, 86). Thus, while bacteriophages may represent P-rich particles in the DOM pool, algal NCVs may represent more N-rich particles in this fraction. This is principally because their N content begins scaling to the cube of the radius as the NCV capsid is filled with DNA as well as proteins. The presence of inner lipid membranes further increases the elemental richness, because lipid head groups are diverse, frequently including P, N, and sulfur (S) moieties (87). The fatty acids of the NCV membrane lipids not only strongly enrich the C content of the viral particle but also increase the energy content, as lipids are among the more reduced, energy-rich biomolecules (88). However, the role of viral particles as an energy source for heterotrophic organisms has yet to be demonstrated. It has been shown in experiments that the breakdown of viral lysates was faster than that of viral particles alone, suggesting they are not as labile (89). Also, when comparing bacteriophage-amended and nonamended cultures, it was reported that viruses may in fact reduce the growth efficiency of at least some heterotrophic bacteria (90), perhaps as a result of the energy required to break down the structural biopolymers that comprise viruses (i.e., DNA and proteins). In this aspect, the presence of reduced, energy-rich C in the form of lipid membranes may be a key feature regarding the palatability of viral particles.

The sizes of NCVs infecting heterotrophic protozoans are significantly larger than those of algal NCVs, which is also reflected in their greater C, N, and P atom counts per virion (Fig. 1A to C). These viruses may have evolved to such large bacterial sizes to be perceived as prey and enhance phagocytic uptake by amoebae (91), a mechanism which could also be true for other heterotrophic protozoa. Additionally, many members of the marine plankton community are mixotrophic, meaning they are able to photosynthesize as well as acquire carbon through the ingestion of particles, a feeding mode that in recent years gained increasing attention (92). Considering that mixotrophy occurs at a higher frequency than previously acknowledged, host-virus coevolution may support increasing viral particle sizes to mimic the host’s prey range and thus increase encounter rates with motile phagotrophic hosts (93). Such an evolutionary strategy may explain the exceptionally large algal virus PkV-RF01 (black diamonds in Fig. 1), which infects mixotrophic marine haptophytes (94). There are, however, additional reasons for an increased viral particle size in certain NCVs, including greater diversity due to increased genetic material or benefits in their ecological niches (57). Nevertheless, as a consequence of such coevolution between a virus and its mixo- or heterotrophic host, the grazing by nonhost organisms will also be facilitated. Choanoflagellates are heterotrophic organisms with a nonselective feeding strategy similar to that of sponges, which have been shown to feed on viral particles by laboratory-based assays (40). Two recent environmental studies using single-cell genomics (35) and metagenomics (66) suggest that enhanced grazing by nonhosts due to host-virus coevolution in choanoflagellates may be more widespread in the marine environment. A positive relationship was found between choanoflagellates and several NCVs, including *Mimivirus*, which have been shown to infect certain marine choanoflagellates (95). These relationships could comprise host-virus and/or predator-prey interactions. A better functional understanding of such reported genome-based associations between viruses and previously unknown nonhost organisms is needed to shed light on their ecological role within the marine plankton community, especially with regard to the hetero- and mixoplankton.

## THE “VIRAL SWEEP” AND ITS IMPORTANCE FOR THE MARINE FOOD WEB

As reviewed above, different organisms have been observed to feed on giant viruses; however, the extent to which these particles may contribute to host nutrition is unexplored. Using *O. dioica* and EhV as a pelagic model system (43, 44), we estimated the C, N, and P contents that predators would gain from viral particle grazing. In this system, the nutritional gain can be 24.2 ng C, 3.8 ng N, and 0.2 ng P individual^−1^ day^−1^ (see Text S1 for calculations). For appendicularians, the reported ingestion of phytoplankton can provide up to 11.4 μg C day^−1^ (96), in which case the contribution of viral particles to the daily dietary C is low (<1%), which has also been determined for sponges feeding on viruses (39). This is similar for N and P if we assume a constant Redfield ratio of 106:16:1 C/N/P (97) in phytoplankton and contribution to nutrition. In nanoflagellates, grazing on viruses was suggested to contribute up to 9, 14, and 28% of C, N, and P, respectively (33), suggesting in smaller predators, viruses have the potential to contribute significantly to host nutrition. However, in the natural environment, many predators will feed on different particles, so the contribution of viruses, while significant, would depend on the prey range experienced by the predator.

**FIG 2:**
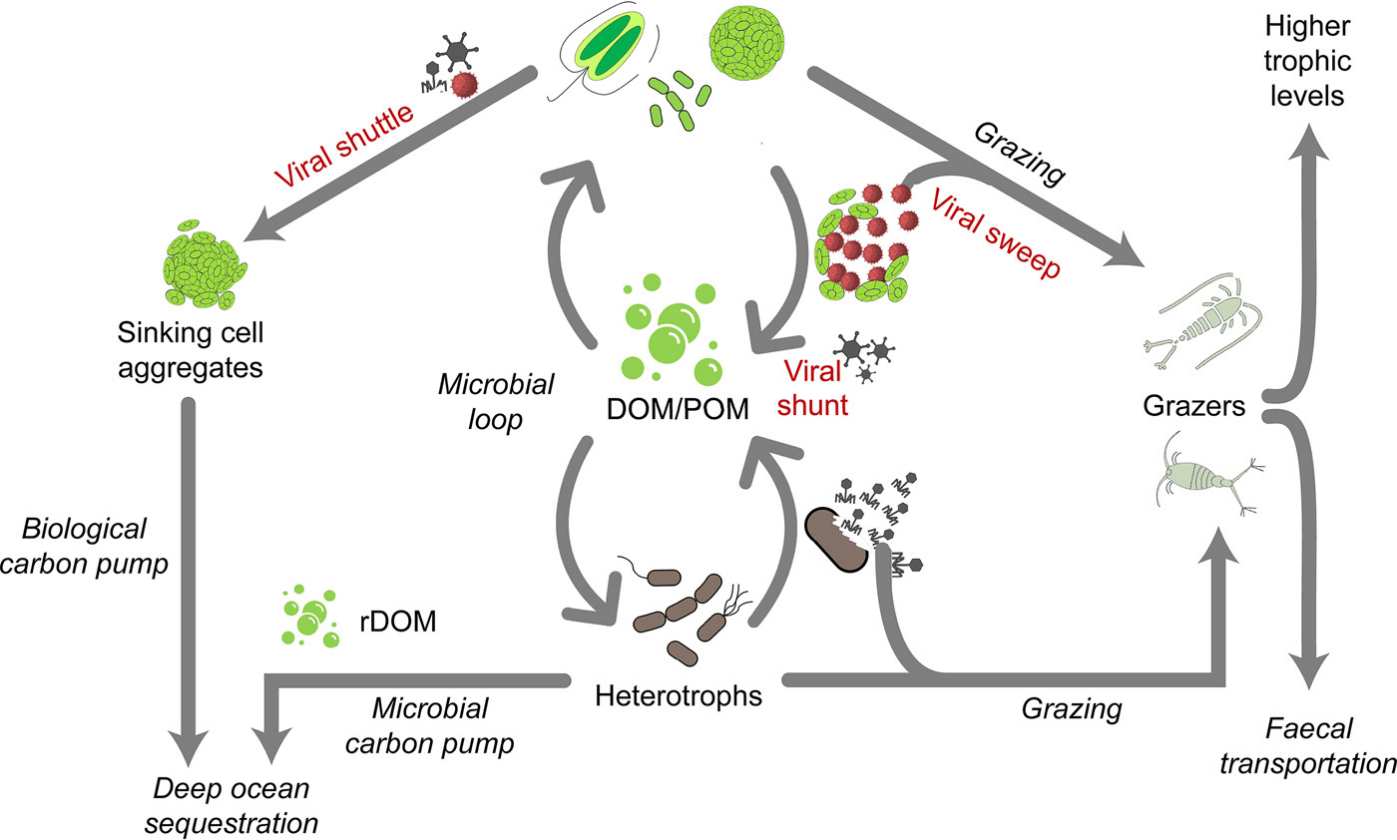
Model of the biogeochemical consequences of viruses in the ocean. Viruses can impact the fate of carbon and other elements within the marine environment in multiple ways. These include the viral shunt (9), which diverts elements from the food web through cell lysis to the marine DOM pool, the viral shuttle (10, 11), which diverts elements from the food web through enhanced aggregation and sinking to the deep sea, and the viral sweep, as highlighted in this review, which diverts elements back into the food web through the ingestion of viral particles. The model was adapted from Kolundžija et al. (137).

Viruses are known to have diverse effects on the fate of C and other elements within the marine environment (Fig. 2). Through the infection and lysis of phototrophic and heterotrophic hosts, they transfer C from the particulate to the dissolved fraction and away from the classical food web, thus making it available to other organisms, such as bacteria (viral shunt) (7, 9). Through the lysis and release of sticky substances (e.g., transparent exopolymers), viruses can lead to increased aggregation of particles and the rapid export of material from the surface to the deep ocean (viral shuttle) (10, 11). In our model, we propose a third mechanism, the “viral sweep,” by which C is diverted back to the classical food web through the ingestion of viral particles by marine grazers. Here, the viral infection of host cells leads to the release of viral particles by means of budding (virus exit by envelopment of its capsid by a cellular membrane) or lysis, which are then grazed upon by different organisms (described above). This would allow the C assimilated from the host cell in the viral particles to be swept back into the classical food web and passed along to higher trophic levels. In the model system presented above, a single *O. dioica* organism can be responsible for 24.2 ng C, 3.8 ng N, and 0.2 ng P individual^−1^ day^−1^. Considering that this organism can form dense blooms (up to 53 individuals L^−1^) (98), with abundances and feeding rates sometimes even exceeding those of copepods (99), this could lead to a significant elemental flow. There are also other pelagic organisms, as well as bottom-dwelling organisms (e.g., sponges) that can contribute to the viral sweep. The relative strength of the viral sweep would depend on several factors, including the size and abundance of viral particles, the predator organisms present and their feeding mechanisms, as well as other factors influencing virus removal, such as UV or chemical inactivation, temperature, or adsorption (31, 32). A current challenge is to quantify the fluxes of viral particles in the marine environment from their sources to their sinks.

## UNPEELING THE VIRAL KIWI—ARE VIRAL PARTICLES DIGESTED?

Whether organisms can digest viruses will have implications not only for the fate of viruses as infectious particles in the marine environment but also for the nutritional gain that a predator can achieve from ingested viral particles. However, experimental evidence on the digestion and assimilation of viruses by marine grazers is scarce. In a recent screening of freshwater ciliates to graze upon viruses, *Halteria* was found to not only ingest the viral particles, but to also grow and divide when consuming chloroviruses as sole food source (100). In two marine zooplankton genera, namely, *Calanus* and *Oikopleura*, fecal pellets were found to contain infectious viruses, suggesting that some viruses can pass through the grazer gut undigested and remain viable (44, 101). Transmission electron microscopy images of fecal pellets and radiolarian food vacuoles from the Ross Sea reported the occurrence of hollow capsids, suggesting at least a partial digestion of viral particles (102). Grazing experiments with nanoflagellates using fluorescently labeled viral particles detected a decline in fluorescence (33), suggesting their effective digestion in these organisms. It is likely that the biochemical conditions within the grazer’s digestive tract will play an important role in the effective digestion of viral particles. For instance, in nanoflagellates, the ingested prey is found in highly acidic food vacuoles, which is more likely to lead to digestion than the passage through the acidic to neutral gut compartments of *Calanus* and *Oikopleura* (103, 104). Recent infectivity experiments on four Antarctic sea ice bacteriophages found a significant reduction in infectious titers at pH 3 and pH 5 (105), which illustrates the role of acidic conditions for the inactivation of viral particles. The gut passage time will further impact the efficiency with which ingested particles are digested and nutrients are assimilated. Zooplankton seem to regulate this time based on various prey parameters, including prey quantity, quality, and digestibility (92, 106, 107). To shed light on the bioavailability of marine viruses, we need a better understanding of the rates of digestion and assimilation of the elements that are present within viral particles. This will further help to determine the relevance of viral particle grazing as a feeding strategy by marine grazers.

## FUTURE PERSPECTIVES

There are many uncertainties regarding the chemical composition of marine viruses, and indeed, many assumptions were made in our calculations. While the nucleic acid and protein contributions can be predicted, the lipid component has to be experimentally assessed. For most membrane-containing viruses, however, the lipid composition is currently unknown. Arguably, the lipid profile of marine viruses is best studied for EhV, an algal NCV that comprises both an inner and outer lipid membrane (65). Interestingly, the reported EhV lipid composition shows some variability (64, 108, 109), which could be a result of differences in infection dynamics, but it could also be a consequence of various relative contributions of extracellular membrane vesicles during sample preparation and lipid extraction. In addition, high lipid content has been described for several algal viruses, including the algal virus PkV RF01, which contains convoluted inner membranes that occupy ~66% of the capsid interior (62). The composition of such lipid membranes will contribute to the C, N, and P contents of viral particles (110). Future research should focus on the lipidomic profiling of other NCVs, particularly as lipids not only influence the biogeochemical composition of virions but also play important roles in the infection dynamics (108, 111–113).

In addition to macronutrients, micronutrients are essential for microbial life within marine environments (114), which may be also contained in viral particles. Recently, Fe has been discovered in the tails of bacteriophages (115), which is an important micronutrient that can limit primary production in vast ocean areas (116). It has been suggested that viral particles may act as organic Fe-binding ligands constituting up to 70% of the Fe attached to organic particles in the surface ocean (115). Also, the trace element zinc, which is an important component particularly of eukaryotic proteomes (117), has been found in the tail proteins of bacteriophages (118). As macro- and micronutrients can limit the productivity of aquatic ecosystems (119), their acquisition from viral particles can alleviate nutrient stress from marine organisms and increase net production. However, whether NCVs contain micronutrients is currently unknown, and the elemental stoichiometry of marine viruses has been primarily assessed by modeling, owing in part to the historical technical challenges in such measurements. Newer analytical approaches that measure elemental and isotopic compositions at the single-cell level using X-ray microanalysis, stable isotope mass spectrometry, and Raman microspectroscopy (120–122), offer promise of more detailed future understanding of the elemental composition of viruses and their role in the cycling of micronutrients, such as iron and zinc, in the marine environment.

Besides the compositional aspects of viral particles, many unknowns remain regarding the ingestion and subsequent digestion of viral particles by marine microbes. The amount of sequencing data of different plankton size classes has significantly increased in recent years due to global-scale ocean surveys, such as the TARA Oceans (123) and Malaspina (124) expeditions. These metabarcoding and metagenomic resources have helped to unravel unknown microbial interactions ranging from predator-prey to host-virus interactions (30, 125) and may similarly assist in revealing novel viral grazers. A challenge thereby is the prediction of the type of association for such frequently cooccurring organisms. In the case of viruses cooccurring with host organisms, the association could range from random adsorption to viral infection to grazing. Supporting evidence may be derived from imaging-based approaches, such as laser scanning confocal microscopy (126) or environmental high-content fluorescence microscopy (127), in combination with labeling techniques for viral DNA or viral proteins (128–130). Recent advancements in the spatial and mass resolution of mass spectrometry technologies, such as nanoSIMS and OrbiSIMS (131, 132), in combination with isotopic labeling may allow for the trophic transfer of viral proteins upon digestion into the grazer’s proteome to be traced. This has been done recently in the context of a host-virus interaction in the lab (133) and for single-metabolite imaging across vesicles with a size of ~200 nm (134), which is in the size range of marine NCVs. In addition, understanding the breakdown of viral capsids and lipid membranes can shed light on the fate of ingested viruses within predators. This could be through tracking the fate of fluorescent viral particles (33), monitoring the grazer’s gene expression (135) and activity of digestive enzymes (e.g., lipases and proteases) (136), or determining if viruses passing through organisms remain infectious (44, 101). Expanding culture-based experiments to different model systems utilizing the techniques discussed above will provide us with necessary information on the strength of the viral sweep, as well as how widespread this mechanism is likely to be. Finally, an important aspect is the consequence of viral particles as a food source on grazer fitness parameters, such as growth rates, secondary production, or fecundity.

Although predation on marine viruses has been known for some time, research on grazing of viral particles by marine microbes is still in its infancy. The increasing body of knowledge on the prevalence of viruses within the marine environment and the current technological advances provide researchers with the tools to fill this fundamental knowledge gap. In light of a future changing ocean, we need to better understand the fate of viral particles by grazing and the relevance of this process, here coined the “viral sweep,” for global biogeochemical cycles.
