# Supplementary material for: Grazing on Marine Viruses and Its Biogeochemical Implications
Source: mBio. 2023 Jan 30;14(1):e01921-21. doi: 10.1128/mbio.01921-21 (PMC9973340; doi:10.1128/mbio.01921-21)
Supplement: TABLE S1 [file mbio.01921-21-s0002.docx]

**Table S1. Reported grazing rates on viral particles by marine organisms.**

| **Grazer** | **Grazer size range** | **Virus (strain)** | **Clearance rate*** | **Reference** |
| --- | --- | --- | --- | --- |
| Appendicularian  *Oikopleura dioica* | 0.99 ± 0.16 mm | *Emiliania huxleyi* *virus 99B1*  (EhV 99B1) | 50 mL^-1^ ind^-1^ d^-1^ | Lawrence et al., 2018 (43) |
| Appendicularian  *Oikopleura dioica* | 0.96 ± 0.15 mm | Emiliania huxleyi virus  (environmental) | 90.3 mL^-1^ ind^-1^ d^-1^ | Mayers et al., 2021 (44) |
| Appendicularian  *Oikopleura dioica* | 0.96 ± 0.15 mm | Large virus  (environmental) | 48.5 mL^-1^ ind^-1^ d^-1^ | Mayers et al., 2021 (44) |
| Breadcrumb sponge  *Halichondria panicea* | 1.76 ± 0.99 g (dry weight) | *Phaeocystis globosa virus 07T* (PgV-07T) | 77 mL^-1^ ind^-1^ h^-1^ | Welsh et al., 2020 (40) |
| Red sea sponge *Negombata magnifica* | Not reported | Virus-like particles  (environmental) | 3.1 x 10^9^ particles d^-1^ | Hadas & Marie, 2006 (39) |
| Nanoflagellates (natural enrichment) | 2-20 µm** | Marine bacteriophage (PWH3a-P1) | 0.3 μL ind^-1^ d^-1^ | Gonzalez & Suttle, 1993 (33) |

*****For *N. magnifica*, ingestion rate was measured instead of clearance rate.

** Size is reported as the operationally defined size range of “nano”
