# Supplementary material for: Grazing on Marine Viruses and Its Biogeochemical Implications
Source: mBio. 2023 Jan 30;14(1):e01921-21. doi: 10.1128/mbio.01921-21 (PMC9973340; doi:10.1128/mbio.01921-21)
Supplement: TEXT S1 [file mbio.01921-21-s0001.docx]

**Supplementary Information**

**Grazing on marine viruses and its biogeochemical implications**

Kyle M. J. Mayers^1^, Constanze Kuhlisch^2^, Jonelle T. R. Basso^3‡^, Marius R. Saltvedt^4^, Alison Buchan^3^, Ruth-Anne Sandaa^4^

^1^ Environment and Climate Division, NORCE Norwegian Research Centre, Bergen, Norway

^2^ Department of Plant and Environmental Sciences, Weizmann Institute of Science, Rehovot, Israel

^3^ Department of Microbiology, University of Tennessee Knoxville, Tennessee, USA

^4^ Department of Microbiology, University of Bergen, Bergen, Norway

Current Address:

^‡^DOE Joint Genome Institute, Lawrence Berkeley National Laboratory, Berkeley, CA, USA

Corresponding author: kyma@norceresearch.no

**Supplementary Text**

*Average molecular formula of a base pair for Nucleocytoviricota (*NCV)*, X_bp_*

In the model of Jover et al. targeting bacteriophages, the average molecular content of a base pair was obtained assuming that the distribution of the nucleobases adenine, thymine, cytosine and guanine in the DNA is uniform leading to an average molecular formula for the base pair of C_19.5_N_7.5_P_2_ [(28)](https://www.zotero.org/google-docs/?bWNatu). They further showed for 776 bacteriophages that the mean frequency of the adenine-thymine pair is 0.52 and that thus their assumption is a good approximation. In the case of NCV, the GC content on average is lower (~0.3)[(131)](https://www.zotero.org/google-docs/?4nrqkN). Furthermore, the presence of modified nucleobases in viral genomes has been reported [(132)](https://www.zotero.org/google-docs/?YNqju0). In our model, we used for simplicity the assumption of Jover et al., which may lead to an overestimation of the nitrate content.

*Filling fraction of DNA inside* NCV *capsids, fill*

The *fill* was calculated following Jover et al. [(28)](https://www.zotero.org/google-docs/?dJtMiI) by fitting the data listed in **Supplementary Table S2** (virus, genome size in kb, internal radius in nm; derived from Chaudhari et al. [(57)](https://www.zotero.org/google-docs/?yI931u)) to the equation n_bp_ = c * (r − h)^3^ obtaining on average c = 0.29 for protozoan NCV and c = 1.3 for algal NCV. The *fill* is then computed using the formula *fill* = $\frac{3vc}{4\Pi}$ to be 0.07 ± 0.04 for protozoan NCV and by 0.33 ± 0.16 for algal NCV. It has to be noted that the filling fraction of NCV does depend on genome size, different to bacteriophages [(57)](https://www.zotero.org/google-docs/?O3KOWL). This means that with increasing genome size the capsid volume increases much more, in particular regarding protozoan NCV, leading to a lowered genome packaging density and lower filling fraction.

*Average atom number of carbon and nitrogen per volume of viral protein, d_C_ and d_N_*

We assumed that the number of atoms of C per unit volume of protein (d_C_) and the number of atoms of N per unit volume of protein (d_N_) are comparable to bacteriophages, and therefore used the values that were computed in Jover et al. based on 2,815 bacteriophage proteins [(28)](https://www.zotero.org/google-docs/?Qch0a1):

d_C_ = 0.73^−1^ * 10^−21^ g/nm^3^ * 2.29 * 10^22^ atoms/g = 31 ± 1 atoms/nm^3^

d_N_ = 0.73^−1^ * 10^−21^ g/nm^3^ * 6.33 * 10^21^ atoms/g = 8.7 ± 0.4 atoms/nm^3^

*Thickness of the* NCV *capsids, h*

Bacteriophage capsids are usually one protein layer thick with an average thickness of 2.5 nm as given in [Jover et al.](https://www.zotero.org/google-docs/?8EQG6l) [(28)](https://www.zotero.org/google-docs/?lmFiYS), or 3 nm as stated in Lošdorfer Božič et al. [(133)](https://www.zotero.org/google-docs/?uYFSL1) and Chaudhari et al. [57)](https://www.zotero.org/google-docs/?adDAzQ). In contrast, the capsids of NCV are much thicker with ~10 nm [(57)](https://www.zotero.org/google-docs/?9hy6lc). Taking into account that NCV are enveloped, this 10 nm thick capsid is composed of a proteinaceous shell as well as a lipid bilayer, the latter being reported to be about 4 nm thick [(134)](https://www.zotero.org/google-docs/?TLKezH). We thus adjusted the parameters to 6 nm for the protein capsid and 4 nm for the lipid membrane. In principle, it would be further possible to account for the difference in the major capsid proteins, as most NCV are known to use the double-jelly-roll fold (7.5 nm thickness)[(135)](https://www.zotero.org/google-docs/?IMnrMb), while tailed bacteriophages mostly seem to use the HK97 fold (1.8 nm thickness)[(136)](https://www.zotero.org/google-docs/?RyEcjy).

*Thickness of the* NCV *lipid membranes, m*

Most NCV contain a single inner lipid bilayer directly below the capsid shell [(57)](https://www.zotero.org/google-docs/?7iFvfs) that may be estimated to approximately 4 nm in thickness based on a dipalmitoyl phosphatidylcholine lipid bilayer [(134)](https://www.zotero.org/google-docs/?evqh93).

*Molecules of carbon, nitrogen and phosphorus per volume of lipid membrane, l_C_, l_N_ and l_P_*

We assumed the number of atoms of C (l_C_), of N (l_N_), and of P (l_P_) per unit volume of lipid membrane based on i) an average surface area per lipid molecule of 0.5 nm^2^ [(74)](https://www.zotero.org/google-docs/?sHGkGH) and a length of 2 nm leading to a volume of 1 nm^3^ per lipid unit, and ii) a simplified membrane composition of only phosphatidylcholines with one 16:0 and one 16:1 fatty acid chain for each lipid molecule (C_40_H_78_O­_8_NP), which are reported to be the most abundant fatty acids in marine algae [(75)](https://www.zotero.org/google-docs/?xrFKTJ). This led us to:

l_C_ = 40 atoms/nm^3^

l_N_ = 1 atoms/nm^3^

l_P_ = 1 atoms/nm^3^

*Calculation of ingested virus stoichiometry of EhV-O.dioica model system*

The number of viral particles ingested per day by *O. dioica* was calculated using clearance rates and abundances in Mayers et al., 2021 (44). Using the average clearance rate of 90.1 and an abundance of 8 x 10^5^ EhV mL^-1^ the number of EhV cleared was 4.03 x 10^7^ viral particles individual^-1^ d^-1^.

To calculate the elemental gain by *O. dioica* from EhV, the number of C, N and P atoms per virion was averaged from the two available strains in NCBI (see **Supplementary Table S2**), EhV-99B1 and EhV-86. The number of atoms was converted to moles by dividing by Avogadro’s constant (6.022 x 10^23^) and multiplying by the molar mass of C, N and P (12, 14 and 30.97 respectively). This was then converted to femtograms C, N or P mL^-1^.
